# Labour monitoring and decision support: a machine-learning-based paradigm

**DOI:** 10.3389/fgwh.2025.1368575

**Published:** 2025-04-16

**Authors:** Mariana Nogueira, Sergio Sanchez-Martinez, Gemma Piella, Mathieu De Craene, Carlos Yagüe, Pablo-Miki Marti-Castellote, Mercedes Bonet, Olufemi T. Oladapo, Bart Bijnens

**Affiliations:** ^1^ Department of Engineering, Universitat Pompeu Fabra, Barcelona, Spain; ^2^ IDIBAPS, Barcelona, Spain; ^3^ Medisys, Philips Research Paris, Paris, France; ^4^ UNDP/UNFPA/UNICEF/WHO/World Bank Special Programme of Research, Development and Research Training in Human Reproduction (HRP), Department of Sexual and Reproductive Health and Research, World Health Organization, Geneva, Switzerland; ^5^ ICREA, Barcelona, Spain

**Keywords:** machine learning, unsupervised learning, maternal health, labour, monitoring, trajectory analysis, language style: British English

## Abstract

**Introduction:**

A machine-learning-based paradigm, combining unsupervised and supervised components, is proposed for the problem of real-time monitoring and decision support during labour, addressing the limitations of current state-of-the-art approaches, such as the partograph or purely supervised models.

**Methods:**

The proposed approach is illustrated with World Health Organisation's Better Outcomes in Labour Difficulty (BOLD) prospective cohort study data, including 9,995 women admitted for labour in 2014–2015 in thirteen major regional health care facilities across Nigeria and Uganda. Unsupervised dimensionality reduction is used to map complex labour data to a visually intuitive space. In this space, an ongoing labour trajectory can be compared to those of a historical cohort of women with similar characteristics and known outcomes—this information can be used to estimate personalised “healthy” trajectory references (and alert the healthcare provider to significant deviations), as well as draw attention to high incidences of different interventions/adverse outcomes among similar labours. To evaluate the proposed approach, the predictive value of simple risk scores quantifying deviation from normal progress and incidence of complications among similar labours is assessed in a caesarean section prediction context and compared to that of the partograph and state-of-the-art supervised machine-learning models.

**Results:**

Considering all women, our predictors yielded sensitivity and specificity of ∼0.70. It was observed that this predictive performance could increase or decrease when looking at different subgroups.

**Discussion:**

With a simple implementation, our approach outperforms the partograph and matches the performance of state-of-the-art supervised models, while offering superior flexibility and interpretability as a real-time monitoring and decision-support solution.

## 1 Introduction

Most pregnancy-related deaths and severe morbidities originate around the time of childbirth, making quality of care during this period critical for positive outcomes (1). The closest to a reference labour monitoring and decision support tool has been World Health Organization (WHO)'s partograph. However, it has failed to fully establish its value, for reasons including its one-fits-all definition of healthy spontaneous progress and overall lacking evidence of positive impact of its use (1). Currently, there is little consensus regarding the best approach to labour monitoring and decision-making, and practice is highly nonstandardised (2), exhibiting some concerning patterns, e.g., significant disparities in Caesarean Section (CS) rates among and within countries, correlating with wealth inequities (3). In this context, the WHO has identified the need for the development of better evidence-based monitoring and decision-support tools. The recent emergence of Machine Learning (ML) represents new opportunities in this regard, and several studies have been focusing on the (supervised) learning of predictive models of CS (4–9). However, most models are not designed for continuous decision support. Souza et al. (10) addressed this limitation by learning different predictive models for different time intervals after the onset of the active phase of labour (“interval models”). There are, however, some limitations to this approach: (1) the intervals had to be large (2 h+) to accommodate enough training data, which limits compatibility with real-time support; (2) only women with available data at exactly the onset of the active phase were eligible (below 30% in their study); (3) higher performances reported for later intervals should be interpreted cautiously, as they apply to a very small subset of women (∼5% in their study, for the latest interval); (4) additionally, later intervals encompass the slowest labours, which may play a role in CS prediction being easier. On the other hand, even the best-performing model has limitations. Due to the highly non-standardised nature of CS practices (3), the occurrence (and prediction) of CS may not always indicate a true risk of adverse outcome–a critical consideration in decision support contexts. Achieving a certain performance threshold also partially depends on the consistency of practices represented within the dataset. For all these reasons, a decision-support system based on “blind” predictions from this type of models alone is unrealistic.

**Figure 1:**
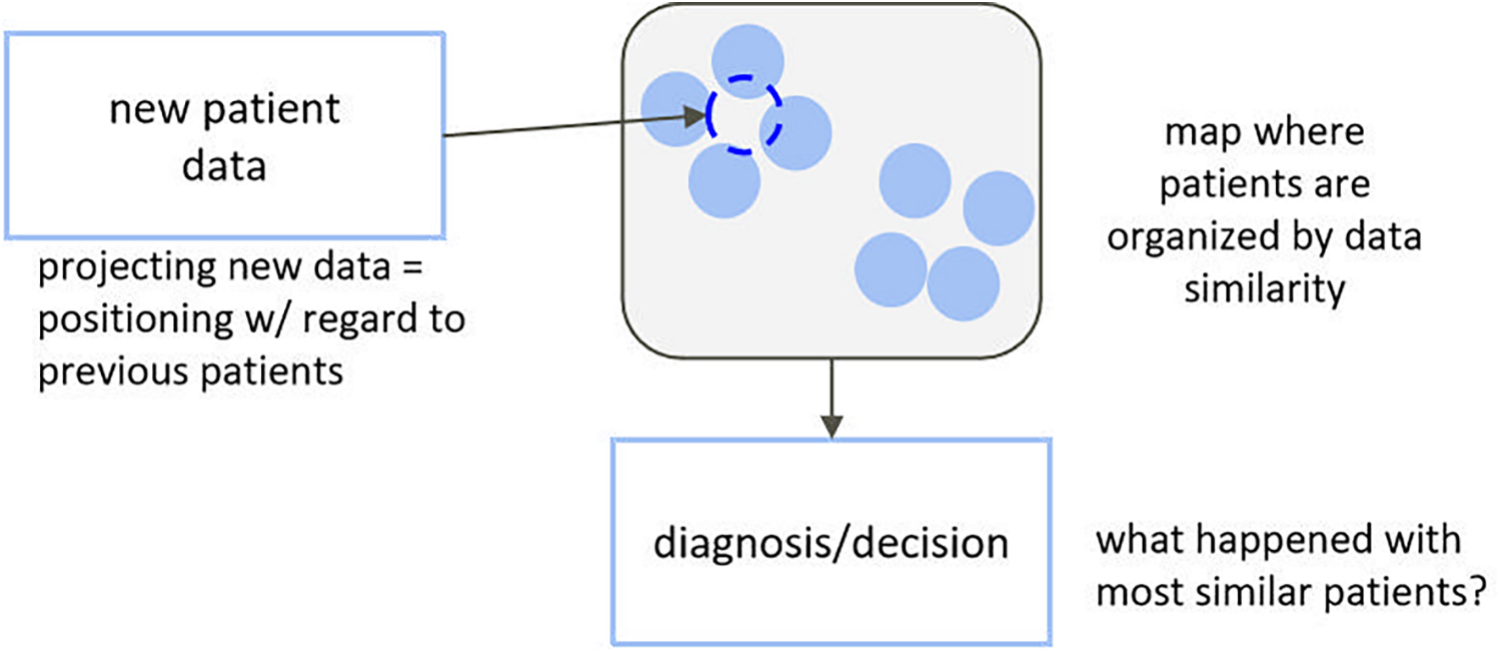
Decision support based on unsupervised dimensionality reduction.

A more prudent and interpretable approach, closer to the traditional way of working of the clinician, might be one based on unsupervised learning, where all complex data are used in an agnostic way to map individuals to a simplified representation where they appear close to each other if they clinically present in a similar way and far from each other otherwise. Clusters of similar subjects can then be identified, and common characteristics (phenotypes) can be described and linked to diagnostics, treatment response, and so forth (Figure 1). Multiple studies have demonstrated the usefulness of this type of approach to support diagnosis or treatment selection (11–20), but they usually explore decision support as a static process and in well-standardized clinical contexts. In this paper, we propose an adaptation of this type of approach for a labour-like context—one that requires continuous and asynchronous monitoring and decision support and does not match the well-standardised nature of clinical trials.

## 2 Methods

### 2.1 Paradigm definition

#### 2.1.1 Fundamentals

**Figure 2:**
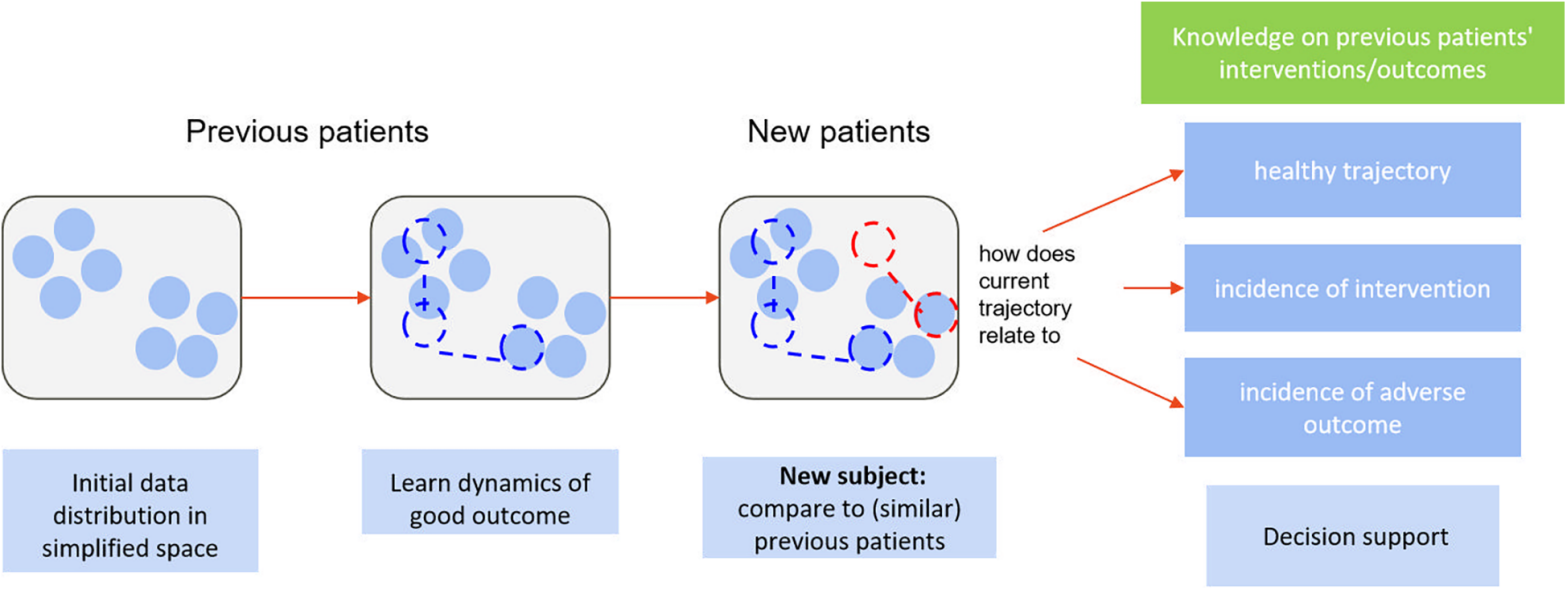
Illustration of the proposed paradigm.

In clinical reality, individual presentation can be very heterogeneous, and one's expected “healthy” trajectory can depend on individual characteristics. With unsupervised dimensionality reduction, we can obtain a representation of initial presentation where subjects are grouped by similar characteristics. To monitor a new subject, their initial data are mapped to said representation—close to the projections of the most similar previous subjects, in terms of all available clinical information, herein referred to as peers. Dynamic changes in subject data translate into dynamic changes in their positioning with regards to each other in the simplified space, defining low-dimensional trajectories. We can then use knowledge, from trials and cohorts, on the temporal trajectories and outcomes of peers to estimate personalised reference healthy trajectories and likelihoods of different events for new subjects. At each follow-up, the new-subject's position in the simplified space and, subsequently, the reference healthy trajectory and likelihoods of important events, are updated. We propose a novel online monitoring and decision-support paradigm that builds upon this line of reasoning, illustrated in Figure 2. This allows transferring the tracking of subject progress from an (often) high-dimensional space onto a simplified and visually intuitive space, to facilitate interpretation (unsupervised component), while also providing dynamic, personalised scores regarding deviation from “normality” as well as likelihoods of important events based on peer knowledge (supervised component). The two components complement each other in supporting decision-making.

Although the development of the proposed methodology was motivated by the specific problem of labour monitoring and decision support, it can generalise to any clinical monitoring and decision-support problem. For that reason, we present it as a generic pipeline (2.1), and subsequently apply it to the specific context of childbirth (2.2).

#### 2.1.2 Implementation

**Figure 3:**
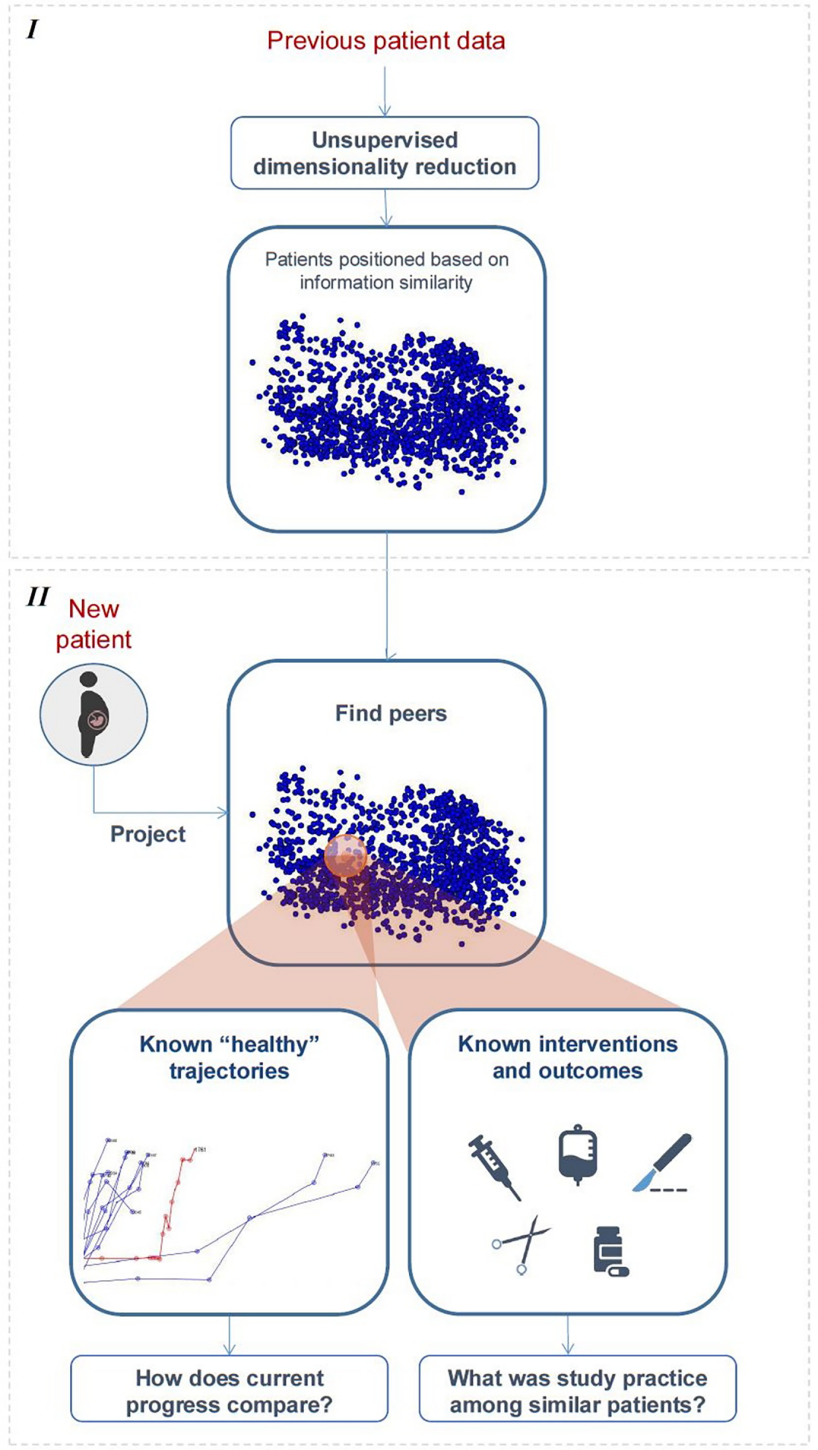
High-level illustration of the proposed framework. Stage I—learning the distribution of “previous subjects” in simplified space. Stage II—peer-based monitoring and decision support.

As Figure 3 illustrates, we first use dimensionality reduction to represent high-dimensional data in a lower-dimensional, interpretable space, where subjects are positioned based on similarities, and temporal data are visualized as trajectories. The monitoring of new subjects is handled by (1) projecting updated data to this space, (2) retrieving peers, i.e., those confined to a close neighbourhood, (3) from those who naturally evolved towards healthy outcomes, estimating a normal/expected progression, and calculating the current subject's deviation from it, and (4) using incidence of interventions/outcomes among peers to compute chance of occurrence for the current subject. Implementation options for this high-level pipeline are diverse; we describe one possible implementation. Herein, a compact description is provided; for a detailed mathematical description, we refer the reader to Supplementary Material Appendix A.

##### 2.1.2.1 Learning distribution of “previous subjects” in simplified space

A necessary first step is learning a projection model to a simplified space from data of “previous subjects” and using it to pre-compute their initial distribution and temporal trajectories (Figure 3, top). In this paper, this was achieved using unsupervised multiple kernel learning (MKL) (11, 12, 14, 21), an algorithm that allows representing heterogeneous features in a unified manner and subsequently merging their information to learn a lower-dimensional embedding of the data where samples are spatially ordered by similarity. The choice of MKL over other linear dimensionality reduction approaches, such as PCA, comes from its ability to address the inherent nonlinearities in labour progression data (22). The preference for unsupervised MKL over other non-linear dimensionality reduction techniques (23) stems from its capacity to integrate heterogenous data features by learning optimal kernel combinations while preserving both local and global data structure.

##### 2.1.2.2 Monitoring new subjects

In this paper, we resort to simple and intuitive methods to illustrate each step of the peer-based dynamic monitoring of new subjects (Figure 3, bottom), based on the model and pre-projected data learned from “previous subjects”.

Given an arbitrary follow-up of a new subject:

1. **Update subject**. The projection model learnt in **(a)** is used to project new data as soon as available, thereby updating the subject's position in the simplified space.
2. **Find peers**. Peers are defined as the “previous subjects” whose projections are in the neighbourhood of the (just-updated) position of the new subject, at the same time since admission. In practice, we define this neighbourhood as a hypersphere centred in said position.
3. **Estimate deviation from ideal progression.** The ideal trajectory is estimated as the average of the temporal trajectories of all peers who experienced uncomplicated, good outcome; the corresponding standard deviation is used to capture healthy variability. In the next follow-up, the subject's new position can be compared to that expected, and a coefficient of “deviation from normality” can be estimated. Specifically, we use the *z*-score (i.e., number of standard deviations away from the expected position).
4. **Predict interventions/outcomes (and timings).** Let us refer to interventions and outcomes as events. We take the proportion of peers that would yet experience a certain event as estimate of chance of its occurrence. Moreover, the distribution of timings of occurrence of a certain event among peers can be used to model chance of occurrence as a function of time.

### 2.2 Application to childbirth

#### 2.2.1 Data

We illustrate the proposed paradigm with the WHO Better Outcomes in Labour Difficulty (BOLD) project dataset (1, 24), including 9,995 labours across 13 Nigerian and Ugandan facilities [see Oladapo et al. (1) for eligibility criteria details]. The primary goal of the BOLD project was to identify the essential elements of intrapartum monitoring that trigger the decision to use interventions aimed at preventing poor labour outcomes. In this project, women characteristics such as demographics, medical history and previous pregnancy information were collected at admission (we refer to these as “static” features), and dynamic maternal and foetal measurements were monitored throughout the course of labour, in nonstandardised time intervals (“dynamic” features). Information on intra- and post-partum complications, interventions and outcomes was also collected. We use 52 features (33 static and 19 dynamic) to characterize women in labour at each moment, detailed in Supplementary Material Tables B.1 and B.2. Feature processing and admission-time data imputation, when performed, are also described in these tables. Missing data among follow-ups was dealt with through previous (follow-up) value propagation. A subset of 549 women who still presented missing data after the described operations was removed from the analysis, as well as an additional 876 women due to time inconsistencies. Experiments were thus performed with data from 8,470 women.

#### 2.2.2 Experiments and analysis

**Figure 4:**
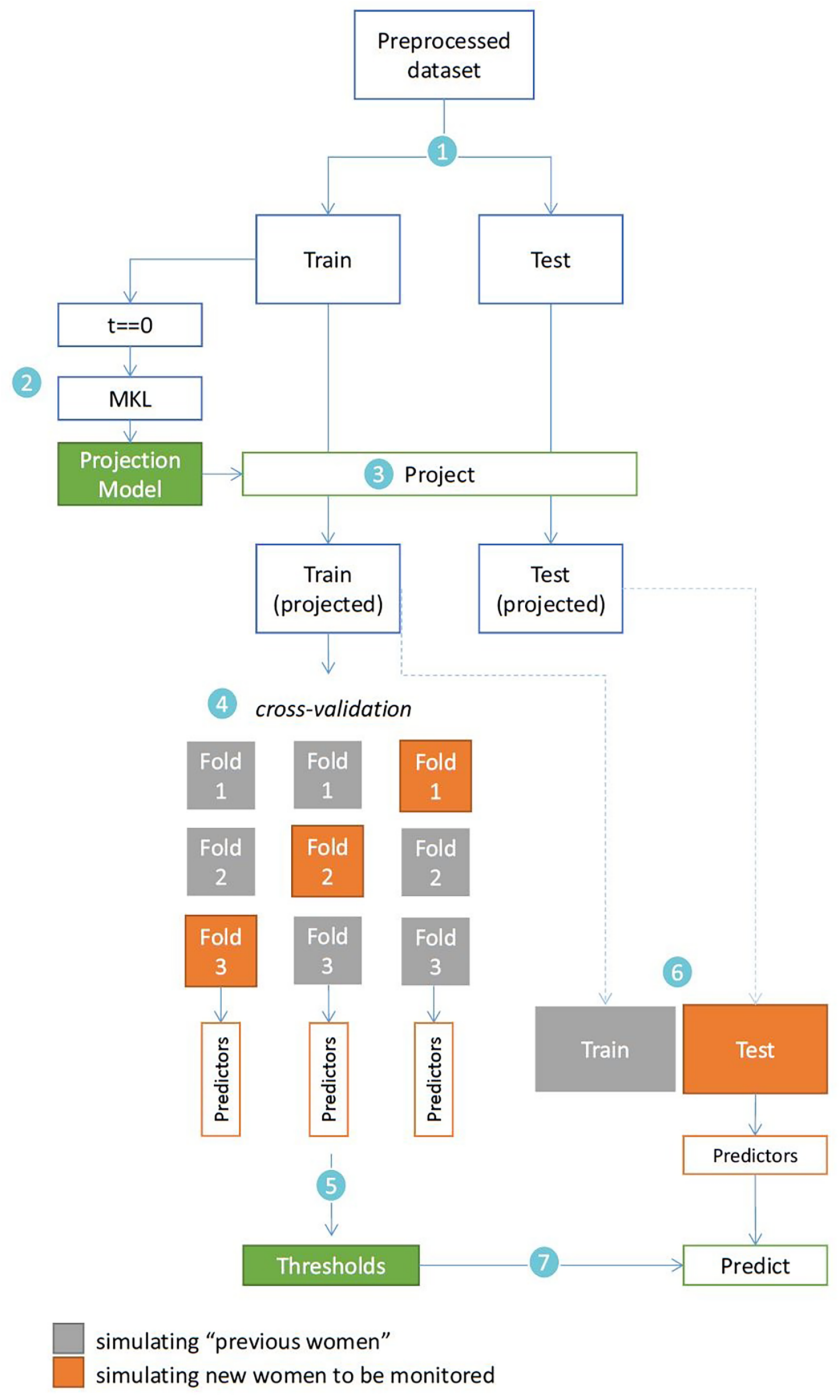
Evaluation of the proposed framework using the BOLD dataset. 1—train/test partition; 2—learning the MKL projection model with the admission-time features of the training set; 3—projecting all training and testing data; 4—three-fold cross-validation and predictor extraction with the training set; 5—extraction of cut-off values for the predictors; 6—framework application and predictor extraction with the testing set; 7—application of learned cut-offs in the testing set predictors.

To illustrate and validate the paradigm, the dataset was randomly separated into training (*n* = 6,349) and testing (*n* = 2,121). Figure 4 illustrates the evaluation of the proposed ML framework using the BOLD dataset. The training set was used to illustrate a “historical cohort of women”, and the testing set was used to simulate new women to be monitored. Uncomplicated labour was defined consistently with previous adverse outcome definitions (25–27).

Two types of analysis are performed: (1) descriptive and qualitative, regarding the interpretability of labour dynamics in the simplified space; (2) quantitative, regarding the supervised component of the paradigm, which uses knowledge on interventions and outcomes among peers to provide risk estimates. For illustration purposes, and given the relevance of this problem in current research, we focus on CS prediction. Three simple, intuitive scores per woman are derived from the paradigm and evaluated as predictors (mathematical description in **A.3**), and subsequently compared to the current state of the art: the maximum values, when considering all follow-ups, of

1. CS chance estimate (according to SELMA study practice, as defined in **2.1.2b-step 4**), $v_{\pi }^{CS}$.
2. Product of chance estimate and the “deviation from normality coefficient” (as defined in **2.1.2b-step 3**), $v_{\pi z}^{CS}$.
3. Product of chance estimate, deviation from normality coefficient and time since admission, $v_{\pi zt}^{CS}$.

For each score, the cut-off that provided the best balance between Sensitivity (SE) and Specificity (SP) was learnt from the training set (using 3-fold cross validation) and subsequently applied to make predictions in the testing set (Figure 4). Performance was compared to those of the partograph's alert and action lines, as well as Souza et al.'s (10) admission and earliest-interval predictive models. Under the assumption that CS practice can be very nonstandardised/biased, we also investigated whether predicting CS was easier for some subgroups of women over others, and whether our approach could be used to aid in the detection and understanding of practice patterns and biases.

## 3 Results

Neighbourhood parameters yielding the results presented in this section are discussed in Supplementary Material Appendix C.

### 3.1 The simplified space and clinical interpretability

**Figure 5:**
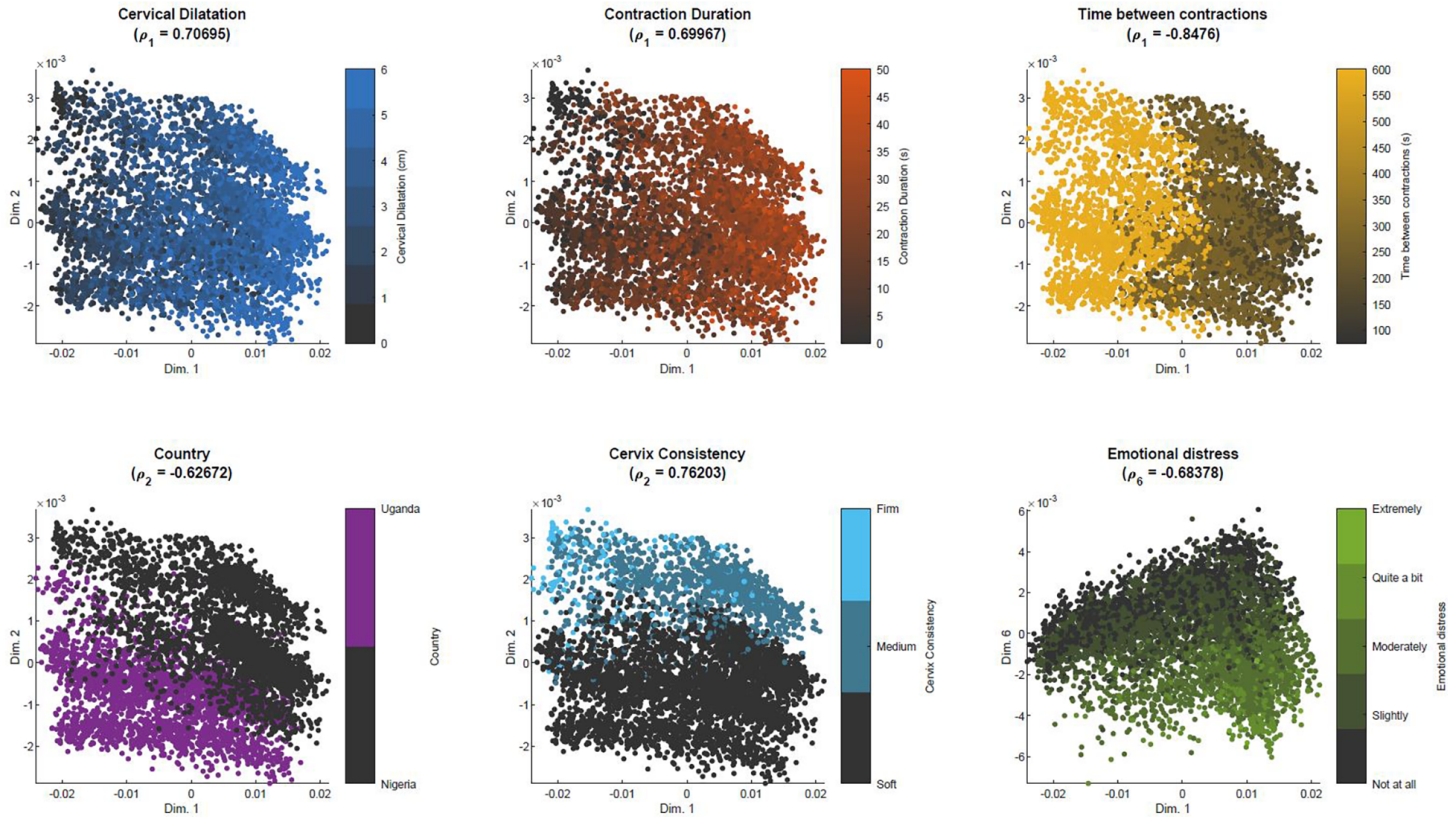
Similarity-based spatial ordering in the MKL space with BOLD dataset. Each plot corresponds to the projections of the samples used to learn the MKL model, color-coded by a specific clinical variable that highly correlates with one of the dimensions of the MKL space. *ρ_d_* = Pearson correlation coefficient between dimension *d* and the clinical variable.

**Figure 6:**
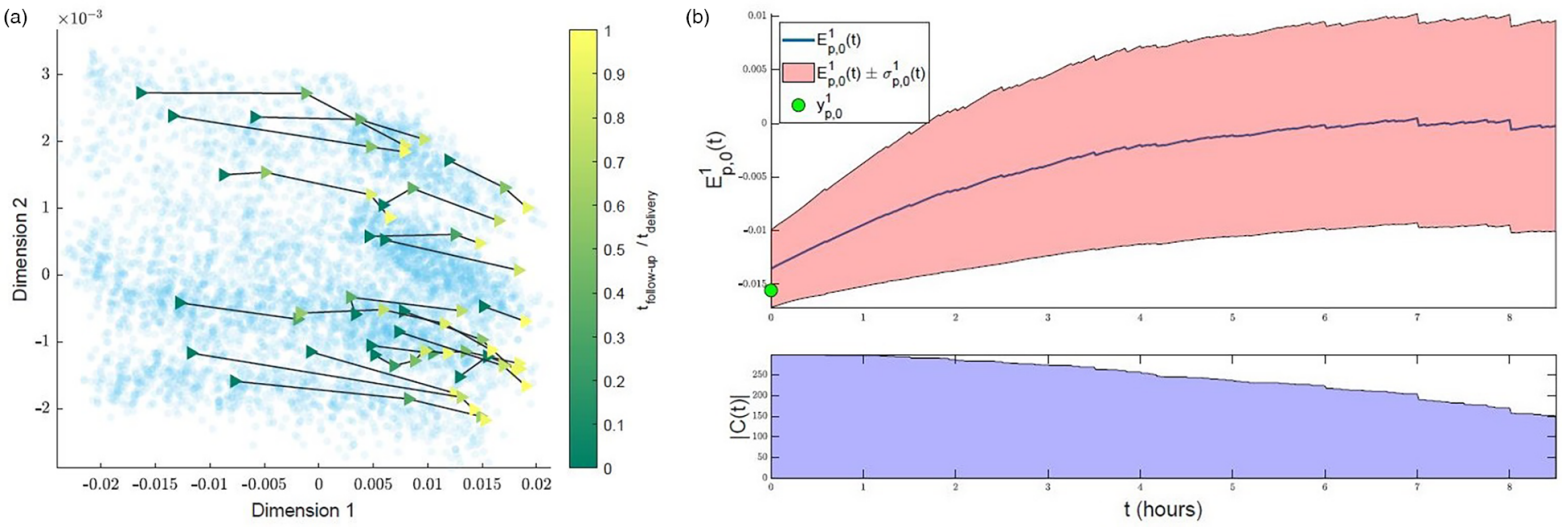
Interpreting trajectories in the MKL space. **(a)** Examples of trajectories defined by training set subjects in the first 2 dimensions of the MKL space. Each sequence of connected triangles corresponds to the trajectory of one subject; the triangles correspond to follow-ups and are coloured by respective follow-up timing normalized by delivery timing (taking admission-time as reference). (b) Example of initial estimate of expected “healthy” progress along the first dimension $\left(E_{\;p,0}^{1}\;(t)\right)$ for a subject with a low initial (first-dimension) value $\left(y_{\;p,0}^{1}\right)$. Top—$E_{\;p,0}^{1}\;(t)$, cropped at the timing where |C(t)| is halved. Bottom—count of the number of peers with uncomplicated labours used to estimate $E_{\;p,0}^{1}\;(t),\;|C(t)|$.

Figure 5 illustrates the initial distribution of the training set (“previous women”) in the simplified MKL space, and its clinical interpretability. Each scatter point corresponds to one woman. As time advances and data are updated, the scatter points (women) move around in the space, defining low-dimensional trajectories. Given that we are dealing with a multidimensional, nonlinear mapping, similarity-ordering in the MKL space can follow complex patterns. For the sake of example, we illustrate cases where clinical variables appear highly ordered along a single dimension of the MKL space, using the Pearson correlation coefficient to identify such cases. The values for all dimension-variable correlation pairs are available in Supplementary Material Figure E.1. Herein, we discuss some of the highest correlations. For instance, the first dimension of the obtained space (Figure 5, top row) strongly correlates with cervical dilatation, duration of contractions and, inversely, with the time between contractions. Thus, in this dimension, women in similar stages of labour are closely positioned, with the leftmost and rightmost regions of the scatter plots mostly populated with women that, at admission-time, were in earlier and later labour stages, respectively. Expectedly, women move towards the right in the scatter plot, as labour advances, as illustrated by Figures 6A,B. Figure 6A overlays the trajectories defined by some of the women of the training set on the admission-time scatter plot of dimensions 1 vs. 2. Each sequence of connected triangles corresponds to the trajectory of one woman, with each triangle corresponding to a follow-up and coloured by its timing normalized by delivery timing. A heterogeneity in initial positioning (i.e., admission-time labour stage) is observed. Nonetheless, all individuals define a rightwards trajectory as labour progresses. In Figure 6B, the initial estimate for “ideal trajectory” (mean in blue ± standard deviation in pink) is plotted for a woman whose initial projection lies on the leftmost region of the scatter plot. As expected, with time, projection values in dimension 1 increase. An initially larger slope gradually decreases, a pattern that is explained by the fact that in the first few hours both slower and faster deliveries are weighing in on the curve estimation, whereas for later timings the remaining slower deliveries push the mean curve down.

The position in the lower-dimensional space is not only dictated by dynamic labour variables. In the bottom row of Figure 5, we can observe that position in dimension 2 correlates with the country variable, while also correlating with cervix consistency, suggesting an association (bias) between country and qualitative assessment of cervix consistency. The rightmost scatter plot suggests that experiencing emotional distress is translated into a downwards displacement in dimension 6.

**Figure 7:**
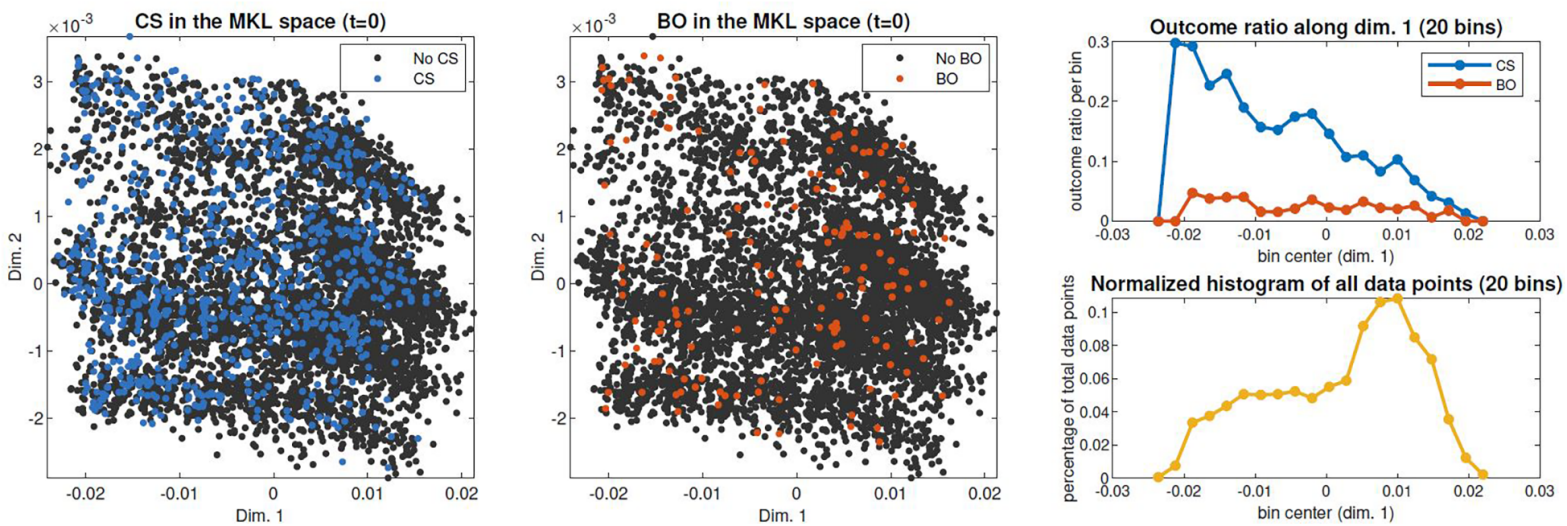
Spatial distribution of outcomes of interest in the admission-time MKL space. Right: CS and bad outcome (BO) rates of occurrence throughout dimension 1, obtained by dividing scatter points in 20 bins along dimension 1 and computing each bin's occurrence rate (top); data density along dimension 1, computed as the percentage of the total scatter points contained in each bin (bottom).

The clinical interpretability of the MKL space can also ease the identification of patterns regarding the occurrence of target events. For example, in Figure 7, analogous scatter plots are generated, this time coloured by the (non)occurrence of CS and adverse outcome. Figure 7-left shows a higher density of CS in subjects on the leftmost region, which we have seen to correspond to earlier-stage labours. This trend is confirmed in Figure 7-right, which displays the outcome ratio along dimension 1. In the case of adverse outcome (Figure 7-center), no evident correlation pattern along dimension 1 is observed. Supplementary Material Figure D.1 extends this analysis to the practice of amniotomy and labour augmentation, with the resulting patterns suggesting a correlation between the incidence of these interventions and initial subject positioning along dimension 2 (Supplementary Material Figure D.1-right). Given the correlation of dimension 2 with country observed in Figure 5, this pattern suggests a higher incidence of both interventions within Nigeria's facilities.

### 3.2 Prediction of CS

Table 1 contains the results of the CS prediction experiments for the complete training and testing populations. During cross-validation, the AUC ranged from 0.746 to 0.767, suggesting a decent predictive power. With the selected cut-offs, our simple predictors largely outperformed the partograph's alert and action lines, achieving a significantly better trade-off between metrics related to the positive (SE, PPV) and negative (SP, NPV) class. For example, $v_{\pi zt}^{CS}$ achieved SE and SP ≈ 0.7, PPV ≈ 0.26 and NPV ≈ 0.94. On the other hand, alert and action lines present relatively good specificity, at the expense of poor sensitivity. When applying the learned cut-offs to the testing set, performances did not significantly change, suggesting good generalizability.

**Table 1:** Cs prediction results.

Train (*n* = 6,349; *n*_CS_ = 817)						
	Th	SE	SP	PPV	NPV	AUC (*p*-value)
Alert line	—	0.540	0.728	0.227	0.915	—
Action line	—	0.290	0.889	0.278	0.894	—
$v_{\pi }^{CS}$	0.221	0.699	0.700	0.256	0.940	0.763 (<0.0001)
$v_{\pi z}^{CS}$	0.422	0.683	0.684	0.242	0.936	0.746 (<0.0001)
$v_{\pi zt}^{CS}$	2.038	0.706	0.707	0.263	0.942	0.767 (<0.0001)
Test (*n* = 2,121; *n*_CS_ = 279)						
	Th	SE	SP	PPV	NPV	AUC (*p*-value)
Alert line	—	0.548	0.731	0.236	0.914	—
Action line	—	0.290	0.891	0.288	0.892	—
$v_{\pi }^{CS}$	0.221	0.674	0.696	0.251	0.934	—
$v_{\pi z}^{CS}$	0.422	0.659	0.712	0.258	0.932	—
$v_{\pi zt}^{CS}$	2.038	0.703	0.712	0.270	0.941	—

*n*, sample size; *n*_CS_, number of positive cases; Th, threshold/cut-off; SE, sensitivity; SP, specificity; PPV, positive predictive value; NPV, negative predictive value; AUC, area under the receiver operating characteristic; *p*-value, fraction of 10,000 random permutation tests for which AUC ≥ AUC observed.

**Figure 8:**
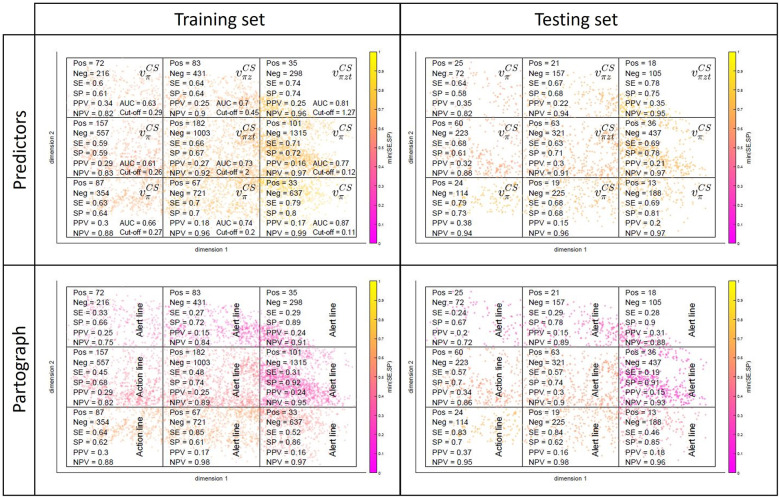
Subgroup performances in the training (left) and testing (right) sets for our predictors (top) and partograph's alert and action lines (bottom).

As mentioned in 2.2.2, because CS practice can be highly non-standardised/biased, we also investigated whether predicting CS was easier for some women subgroups, and whether our approach could help detecting and understanding practice patterns and biases. Figure 8 illustrates the partitioning of the admission-time MKL space in different spatial regions, which is equivalent to splitting women in subgroups of similar characteristics upon admission. The same spatial division is carried out for the training (left) and testing (right) sets. The cut-offs of Table 1 were recomputed for each training subgroup and applied to make predictions in the testing counterparts. In the top rows, the predictor among $\left\{v_{k}^{CS}\},\;k\in \{\pi ,\;\pi z,\;\pi zt\right\}$, with the best performance in testing [measured as the maximum value for min(SE,SP)] is identified for each region, along with the selected cut-off and corresponding performance metrics. In the bottom rows, the process is repeated for the partograph's alert and action lines. All scatter plots are coloured by the subgroup minimum between SE and SP.

The top-left plot of Figure 8 reveals a rightward-oriented gradient in performance, suggesting that the prediction of CS is more effective for subgroups corresponding to later labour stages at admission time. If we merge early- and late-admission women into two larger subgroups (in practice, we separated them in latent- and active-phase labours, based on the 4 cm dilatation threshold recommended at the time the SELMA study was conducted), this effect is evident (see Supplementary Material Table F.1). The gap between the two subgroup performances is significant, with both growing apart from the average performances of Table 1, but in opposite directions—towards poorer performances in the early-admission group ($v_{\pi zt}^{CS}\;$: AUC = 0.646, SE = 0.606, SP = 0.609) and higher performances in the late-admission group ($v_{\pi zt}^{CS}\;$: AUC = 0.813, SE = 0.739, SP = 0.740). Note that CS is approximately twice as incident in the early-admission subgroup, which has a direct effect on PPV and NPV. Note also that, despite the diverging pattern in the two subgroup performances, subgroup-level performance is in both cases being optimised by using subgroup- rather than globally estimated cut-offs (Supplementary Material Figure F.1). It is observed that, to optimize performance, cut-off values increase for the early- and decrease for the late-admission subgroup (Supplementary Material Table F.1), when compared to the globally estimated (Table 1), a result that is not counter-intuitive given the corresponding variations in CS incidence and the latter's role in the very definition of the predictors. Regarding generalizability, in most cases (both in Figure 8 and Supplementary Material Table F.1), subgroup performances in the testing set are comparable to those in the training set. For a more detailed breakdown of the model's performance by subgroups, which are driven by demographics and clinical characteristics at admission time, we refer the reader to Figure 5 and Supplementary Material Figure E.1.

When it comes to the partograph's alert and action lines (bottom row of Figure 8, Supplementary Material Table F.1), subgroup performances range from comparable to significantly worse than those of our predictors, depending on the women subgroup at hand. It is further observed in the bottom row of Figure 8 that they generally perform best in partitions where Uganda is the dominant country (revisit Figure 5), suggesting that in Uganda CS practice was more aligned with the partograph's guidelines.

**Figure 9:**
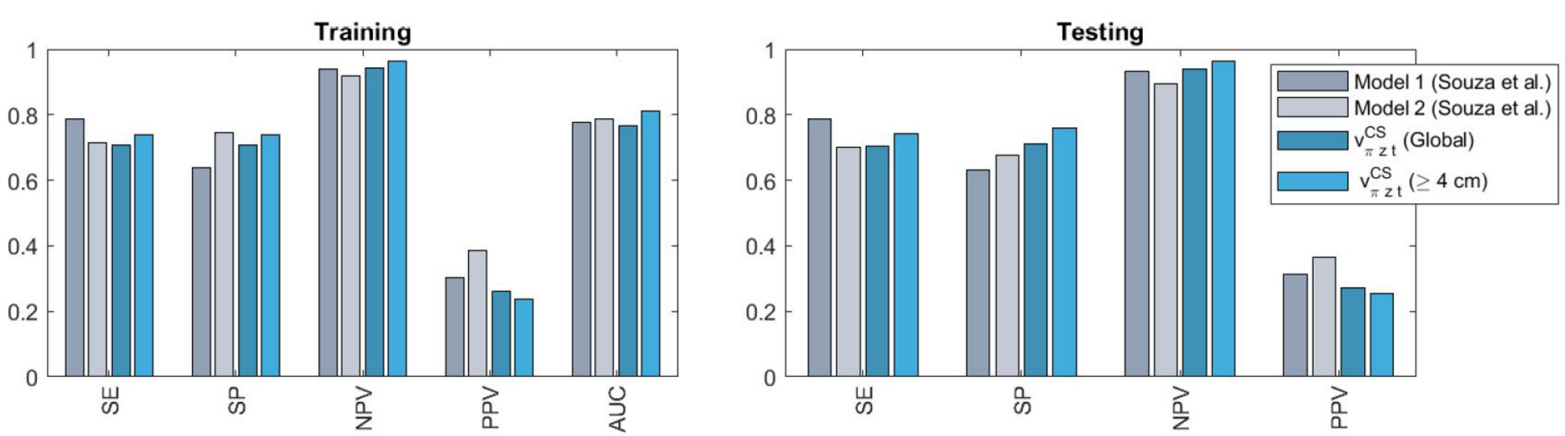
Comparison of the performances obtained with our model $v_{\pi zt}^{CS}$ (on the entire cohort or when considering only late-admission women, defined as ≥4 cm dilatation) against those of admission-time (model 1) and earliest interval (model 2) models by Souza et al. (10). SE, sensitivity; SP, specificity; PPV, positive predictive value; NPV, negative predictive value; AUC, area under the receiver operating characteristic.

Lastly, Figure 9 positions our predictor $v_{\pi zt}^{CS}$, in terms of performance, with regard to the admission-time and earliest interval (0–2 h after onset of 4 cm of cervical dilatation) models by Souza et al. (10) (referred to as Model 1 and Model 2 in the original publication, respectively; also trained and tested with the BOLD dataset). Comparisons are restricted to Models 1 and 2, since sample sizes drop sharply for later interval Models (3 and 4), making comparisons more problematic. It includes the performance of $v_{\pi zt}^{CS}\;$ as in Table 1 and also when considering only late-admission women (Supplementary Material Table F.1). For all models, cut-off values that provided the best balance between sensitivity (SE) and specificity (SP) during training were subsequently applied to make predictions in the testing set. Our simple predictor is observed to perform comparably to the predictive models but can be applied to all individuals and during real-time follow-up, as opposed to Souza's approach.

## 4 Discussion

### 4.1 Main findings

We have presented a novel machine learning-based approach for interpretable, continuous labour monitoring and decision-support. The unsupervised component of the proposed paradigm allows the dynamic visualisation of high-dimensional labour data as low-dimensional trajectories in a clinically-interpretable simplified space, and their comparison with personalised (and also dynamically-updated) estimates of healthy trajectories (Figures 5, 6). This simplified representation also proved helpful in the identification of practice biases—e.g., in the cases of qualitative assessment of cervix consistency (Figure 5) or interventions by amniotomy and labour augmentation (Supplementary Material Figure D.1) between countries, or in the intervention by CS between early and late-admission women (Figure 7). Simple supervised peer-based scores, quantifying deviation from normal progress or likelihood of important events, significantly outperformed the current reference monitoring and decision-support tool (the partograph) and performed comparably to state-of-the-art predictive models, while having wider clinical applicability (Figure 9). Finally, adjusting predictor cut-offs to maximise subgroup-level performances, confirmed our hypothesis that in some women subgroups CS prediction is significantly easier than in others (Figure 8, Supplementary Material Table F.1, Figure F.1).

### 4.2 Interpretation

The differences found regarding the intervention by CS between early and late-admission women align with findings from previous observational studies (28–32). Performances in the problem of CS prediction were overall moderate. As explained in the Introduction, they are also intrinsically limited by the inconsistency of CS practice in the available datasets. Nonetheless, using a straightforward implementation and simple predictors, we could significantly outperform the partograph and perform comparably to previous predictive models. In summary, our paradigm combines attractive features of the partograph (visual assessment, intuitive, interpretable) with performance levels of purely supervised ML models (in this case regarding CS), while overcoming limitations of both approaches (non-personalised, non-dynamically-updated and univariate reference trajectories in the partograph; temporal resolution and applicability limitations in the predictive models). On the other hand, the finding that for some women subgroups CS prediction is significantly easier than in others is likely a direct consequence of CS practice heterogeneity itself—eventually more consistent, thus predictable, in some cases than others. Subgroup analysis can thus be useful to locally optimise predictive performances and to help identifying and understanding practice differences and biases, an important step in terms of the objectives of practice standardization and optimization of intervention towards risk minimization.

### 4.3 Contextualization with existing literature on ML-based labour prediction

When comparing our work to other existing literature, results can vary significantly depending on study population characteristics, cohort size, the parameters included, and the complexity of the ML models used. For example, a study carried out in Iceland (33), found that interrogating transabdominal and transperineal ultrasound data using a Cox Regression model achieved an AUC for prediction of spontaneous delivery of 0.68 (95% confidence interval, 0.55–0.80). Another study from Canada (34), achieved an AUC of 0.77 (0.71–0.82) when predicting emergency caesarean section deliveries based on antenatal obstetric and non-obstetric factors (acquired before the onset of labour) using a multivariate logistic regression model. Finally, a study from the US (35), achieved and AUC of 0.82 at predicting vaginal delivery at 4 h from admission interrogating intrapartum data using a supervised ML model. Despite variations in study settings, datasets, and statistical or ML models used, it is notable that all predictions fall within an AUC range of [0.68–0.82]. This places our model's performance on par with these other ML-based implementations. Furthermore, our model utilizes data that can be feasibly collected in low- and middle-income countries (LMICs), unlike other studies that rely on cardiotocography (36) or ultrasound data (33), which are challenging to obtain in resource-limited settings.

### 4.4 Clinical implications of our study

To emphasize the clinical implications of our study, we have implemented a prototype of a decision support system based on the ML approach presented in this manuscript. Further details of this prototype can be found in Supplementary Material Appendix G. The prototype proposed offers a real-time decision support tool that guides clinicians in monitoring labour progression by providing dynamic risk assessments and intervention recommendations based on a patient's ongoing trajectory. These personalized insights could be particularly relevant in LMICs, where deviations from normal labour can go undetected due to limited access to skilled healthcare professionals. In these settings timely interventions can significantly impact maternal and neonatal outcomes. By offering clear, actionable recommendations, our prototype could support early intervention and better resource allocation, potentially reducing the burden of preventable complications such as prolonged labour or the unnecessary use of caesarean sections.

### 4.5 Limitations

A first limitation is the inherent difficulty in validation—in terms of the prediction of actual risk of adverse outcome/“necessary” interventions—as we only have knowledge on what interventions were performed and the resulting outcomes, but no guarantee of causality. A second limitation relates to the specific implementation of the proposed paradigm, where, for sake of illustration, a simplified version was tested, especially when it comes to the supervised component's estimation of the “ideal” trajectory and risk estimates. This paper thus represents a proof of concept, where simple implementation choices already show the potential of the proposed paradigm, based on the internal validation results. Before clinical integration, a more sophisticated implementation would be required. The dataset, originating from the BOLD project conducted in 2014–2015, may seem dated in comparison to newer datasets available in the literature. However, our methodological approach is designed to be flexible and adaptable to other datasets originating from different contexts such as (37), a more recent cohort from Uganda comprising 1,040 deliveries, or (38), a cohort from Kenya comprising 1,164 deliveries. Furthermore, the BOLD dataset focuses solely on practices in two countries. Additional external validation is required to assess its applicability in other healthcare settings with different population characteristics and clinical practices. It should be stressed that the usefulness of the proposed approach is maximised when the training data are representative of the population and context under study. Finally, the subgroup analysis presented in Figure 8 helps identify potential performance biases or limitations in the predictive model, which could guide future refinements to ensure that our approach benefits a diverse patient population.

## Data Availability

The data analyzed in this study is subject to the following licenses/restrictions: Data sharing conforms to the data use policy governing the BOLD study. Requests for data use should be addressed to the World Health Organization. Requests to access these datasets should be directed to OLADAPO, Olufemi Taiwo (oladapoo@who.int).
