# Supplementary material for: Labour monitoring and decision support: a machine-learning-based paradigm
Source: Front Glob Womens Health. 2025 Apr 16;6:1368575. doi: 10.3389/fgwh.2025.1368575 (PMC12040997; doi:10.3389/fgwh.2025.1368575)
Supplement: Supplementary file 1 [file Datasheet1.pdf]

Supplementary Material

Appendix A: Mathematical Descriptions

A.1 Overview of MKL

Let us consider N data samples, each described by M uni- or multidimensional features. An MKL projection to a D-dimensional space is parameterized by a projection matrix $A \in \mathbb{R}^{N \times D}$ and a vector $\beta \in \mathbb{R}^{M}$ that contains the weight of each feature in the mapping. Instead of operating directly on the raw data, A and β operate on kernelized (similarity) data. Let x_i^m denote the data associated with the mth feature of the ith data sample, with $i = 1, \dots, N$ and $m = 1, \dots, M$. In this paper, all features are unidimensional, so $x_i^m \in \mathbb{R}$. Additionally, let us use the simplified notations $x^m \in \mathbb{R}^N$ for the vector of values of feature m for all N samples, $x^m = (x_1^m, \dots, x_N^m)^T$, and $x_i \in \mathbb{R}^M$ for the vector of M feature values of sample i, $x_i = \left(x_i^1, \dots, x_i^M\right)^T$. Different data types may be associated with different notions of similarity. In this paper, we adopt the kernel functions proposed in [31] for clinical data. Let k^m denote the kernel function associated with feature m. For continuous/ordinal variables, the similarity between input samples i and j is measured by

$$k^m\left(x_i^m, x_j^m\right) = 1 - \frac{\left|x_i^m - x_j^m\right|}{\max x^m - \min x^m} \quad , \qquad (A1)$$

whereas for nominal variables,

$$k^m\left(x_i^m, x_j^m\right) = \delta\left(x_i^m - x_j^m\right) \quad , \qquad (A2)$$

with δ the Kronecker delta function.

Let $K \in \mathbb{R}^{N \times N \times M}$ denote the three-dimensional matrix whose entries are $K_{ijm} = k^m(x_i^m, x_j^m)$. with $i, j = 1, \dots, N$ and $m = 1, \dots, M$. Let $K_i \in \mathbb{R}^{N \times M}$ denote the ith slice of K along the first dimension,

$$K_i = \begin{pmatrix} k^1\left(x_1^1, x_i^1\right) & \cdots & k^M\left(x_1^M, x_i^M\right) \\ \vdots & \ddots & \vdots \\ k^1\left(x_N^1, x_i^1\right) & \cdots & k^M\left(x_N^M, x_i^M\right) \end{pmatrix}, \qquad (A3)$$

and $K^m \in \mathbb{R}^{N \times N}$ the mth slice along the third dimension

$$K^m = \begin{pmatrix} k^m(x_1^m, x_1^m) & \cdots & k^m(x_1^m, x_N^m) \\ \vdots & \ddots & \vdots \\ k^m(x_N^m, x_1^m) & \cdots & k^m(x_N^m, x_N^m) \end{pmatrix}, \qquad (A4)$$

In short, K_i encodes the similarity coefficients among sample i and all other samples (rows), in terms of all M features (columns). On the other hand, K^m is a symmetric matrix that encodes the pairwise similarities of all N samples according to feature m.

In our unsupervised MKL model, the projection $y_i \in \mathbb{R}^D$ of $x_i \in \mathbb{R}^M$, with $D \leq M$, becomes a function of K_i:

$$y_i = A^T K_i \beta \quad . \tag{A5}$$

The MKL problem is then formulated as

$$\min_y \sum_{i,j} \|y_i - y_j\|^2 W_{ij} \quad , \tag{A6}$$

$$s.t. \sum_i \|y_i\|^2 W'_{ii} = 1 \ , \tag{A7}$$

where W is an affinity matrix, computed as a (linear or non-linear) combination of all $\{K^m\}_{m=1}^M$. Thus, each entry W_{ij} encodes the similarity between samples i and j based on contributions from all features. In this paper, W is computed as the average of all K^m. The minimization imposes that samples that are similar in the input space (high W_{ij}) are mapped to close positions in the output space. Constraint **A7** removes an arbitrary scaling factor in the output embedding and eliminates trivial solutions, with $W'_{ii} = \sum_j W_{ij}$. Plugging **A5** into **A6** and **A7** the problem translates into finding A and β such that

$$\min_{A,\beta} \sum_{ij} \|A^T K_i \beta - A^T K_j \beta\|^2 W_{ij} \quad , \tag{A8}$$

$$s.t. \sum_i \|A^T K_i \beta\|^2 W'_{ii} = 1 \ . \tag{A9}$$

In practice, A and β are found by iteratively solving a generalized eigenvalue problem for A, and a semidefinite programming problem for β [32]. Once the model parameters (A, β) are estimated, the projection $y_u \in \mathbb{R}^D$ of a new sample $x_u \in \mathbb{R}^M$ amounts to a generalization of eq. **A5**:

$$y_u = A^T K_u \beta \quad , \tag{A10}$$

where $K_u \in \mathbb{R}^{N \times M}$ and $K_{ium} = k^m(x_i^m, x_u^m)$, with $i = 1, \ldots, N$ and $m = 1, \ldots, M$.

For a more detailed description of the algorithm, we refer the reader to [32].

A.2 Proposed Methodology

We use unsupervised MKL to learn the projection model from data of "previous subjects" and obtain their initial distribution and temporal trajectories in the simplified space. Let us consider we have admission and follow-up data on P "previous subjects" at our disposal. That is, each subject p (with $p = 1, \ldots, P$) has a sequence of follow-ups $f = 0, \ldots, F_p$, with $f = 0$ the first assessment (admission) and F_p the total number of follow-ups. Let t_f^p denote the timing of follow-up f of subject p, computed as the absolute time since admission ($t_0^p = 0$), x_{p,t_f^p} the corresponding data sample, and y_{p,t_f^p} the corresponding MKL projection.

Given a new subject q at follow-up f:

1. **Update subject.** That is, project the data sample $x_{q,t_f^q} \in \mathbb{R}^M$ to $y_{q,t_f^q} \in \mathbb{R}^D$ using eq. **A10**, i.e., $y_{q,t_f^q} = A^T \; K_{q,t_f^q} \, \beta$.
2. **Find peers.** Peers are defined as the "previous subjects" whose projections at time t_f^q are within a limited neighborhood of y_{q,t_f^q}. In practice,
    - For each subject $p = 1, \ldots, P$, retrieve the projection at time t_f^q, y_{p,t_f^q}. Since many "previous subjects" will not have follow-up data specifically at t_f^q, in those cases the value of y_{p,t_f^q} is obtained by linear interpolation on the precomputed y_{p,t_f^p}, $f = 0, \ldots, F_p$.
    - Individual p is considered a peer of q at time t_f^q if

$$\sum_{d=1}^{L} \left(y_{p,t_f^q}^d - y_{q,t_f^q}^d \right)^2 \leq R^2 , \quad L \leq D, \qquad (A11)$$

    i.e., if the projection of subject p is contained within a hypersphere of dimensionality L and radius R centered on that of subject q. To account for scaling differences among dimensions d, condition **A11** is computed on a standardized form (zero mean and unit standard deviation for all dimensions) of the projection data.

3. **Estimate deviation from ideal progression.** Let $\mathbb{H}$ denote the set of peers of subject q at time t_f^q, i.e., the subset of "previous subjects" obeying **A11**. Additionally, let $\mathbb{S}$ represent the subset of the P subjects who progressed spontaneously towards ideal outcome, i.e., without any complications or interventions.

    - The t_f^q update of the estimate of normal progress for time $t > t_f^q$, $E_{t_f^q}(t)$, is given by

$$E_{t_f^q}(t) = \frac{1}{|\mathbb{C}(t)|} \sum_{p \in C(t)} y_{p,t} \ ,$$

$$C(t) = \{p \mid (p \in \mathbb{H} \cap \mathbb{S}) \ \wedge \left(t_{F_p}^p \geq t\right)\} \qquad (A12)$$

corresponding to the mean of the projections of all peers with normal course at time t (provided they exist or can be interpolated for such timing). The corresponding standard deviation $\sigma_{t_f^q}(t)$ is computed as an estimate of "normal" variability.

- In the next follow-up, at time t_{f+1}^q, we can verify how much the projection y_{q,t_{f+1}^q} deviates from the predicted "normal" position, $E_{t_f^q}(t_{f+1}^q)$. Specifically, for each dimension d, we quantify deviation from normality as the z-score

$$z_{t_{f+1}^q}^d = \frac{y_{q,t_{f+1}^q}^d - E_{t_f^q}^d(t_{f+1}^q)}{\sigma_{t_f^q}^d(t_{f+1}^q)} \quad . \qquad (A13)$$

4. **Predict interventions/outcomes (and timings).** Let us refer to interventions and outcomes as events. Let e denote an event of interest, $\mathbb{E}$ the set of "previous subjects" that experienced that event and $t^{p,e}$ the timing of such event for subject $p \in \mathbb{E}$.

- The chance of e, at time t_f^q, is updated as

$$\pi_{t_f^q}^e = \frac{|\mathbb{G}|}{|\mathbb{H}|} \ , \quad \mathbb{G} = \{p \mid (p \in \mathbb{H} \cap \mathbb{E}) \ \wedge (t^{p,e} \geq t_f^q)\} \qquad (A14)$$

i.e., the ratio of peers that experienced e at $t \geq t_f^q$.

- Moreover, a probability density function can be fitted to the distribution of all $t^{p,e}, p \in \mathbb{G}$, so as to obtain an estimate of the probability of e with respect to time, $\pi_{t_f^q}^e(t), \ t \geq t_f^q$.

A.3 Predictors/scores

1. Chance estimate (according to SELMA study practice):

$$v_\pi^{CS} = \max_f \pi_{t_f^q}^e \ , \qquad f = 1, \ldots, F_q \quad . \qquad (A15)$$

2. Combination of chance estimate with deviation from normality:

$$v_{\pi z}^{CS} = \max_{f}\left[\pi_{t_f^q}^{e} * \max_{d}\left|z_{t_f^q}^{d}\right|\right] \quad , \qquad f = 1,\ldots,F_q\,, \quad d = 1,\ldots,D \quad . \tag{A16}$$

3. Combination of chance estimate with deviation from normality and time since admission:

$$v_{\pi z t}^{CS} = \max_{f}\left[\pi_{t_f^q}^{e} * \max_{d}\left|z_{t_f^q}^{d}\right| * t_f^q\right] \quad , \qquad f = 1,\ldots,F_q\,, \quad d = 1,\ldots,D \quad . \tag{A17}$$

Appendix B: Static and dynamic features

Table B.1. *Admission-only / static features. Bold name indicates that the feature appears modified when compared to the original SELMA dataset.*

Name	***Notes***
Country code	Country code: Uganda/Nigeria (1/0)
Ethnicity	Ethnicity: [NIGERIA] 1 - Ibo; 2 - Yoruba; 3 - Hausa; 4 - Fulani; 5 - TIV; 6 - Kanuri; 7 - Other Nigerian; 8 - Non Nigerian; [UGANDA] 9 - Muganda/Musoga/Mugisu; 10 - Munyakore/Mukiga/Munyoro/Mutoro; 11 - Acholi/Langi/Alur; 12 - Iteso/Karamojong; 13 - Lugbara/Madi; 14 - Other Ugandan; 15 - Non-Ugandan
Facility code	1-13
Age	years
Height	cm
Foot length	cm
Current weight	kg
Marital status	Marital status: 0 - Single / Separated / Divorced / Widowed; 1 - Married / Cohabitating
Education level	Education level: 0 - No education; 1 - Other (e.g., Quranic / Nomadic education only); 2 - Pre-primary education; 3 - Incomplete primary education; 4 - Complete primary education; 5 - Incomplete secondary education; 6 - Complete secondary education; 7 - Incomplete post-secondary/tertiary education; 8 - Complete post-secondary/tertiary education
Gainful occupation	Gainful occupation: 0 - No; 1 - Yes
Parity	Number of previous births
Previous abortions or stillbirths	Previous abortions or stillbirths: 0 - No; 1 - Yes
Previous uterine surgery	Previous uterine surgery (includes previous C-sections or other uterine surgeries): 0 - None; 1 - One; 2 - More than one
Best estimate of gestation	weeks

Mode of labour onset and referral (or not) from another health facility	Mode of labour onset and referral (or not) from another health facility: 0 - spontaneous onset, not referred from another facility; 1 - induced, not referred; 2 - spontaneous, referred; 3 - induced, referred
Fetal movements in the last 2h	Fetal movements in the last 2h: 0 - reduced or absent; 1 - no changes/increased
Preterm rupture of membranes	Preterm rupture of membranes: 0 - No; 1 - Yes
Obstetric haemorrhage	Placenta praevia, accreata increta percreta, placentae abruption or other obstetric haemorrhage: 0 - No; 1 - Yes
Pre-eclampsia or eclampsia	Pre-eclampsia or eclampsia: 0 - No; 1 - Yes
Cervix effacement	Cervix effacement: 0 - Thick (less than 30% effaced); 1 - Medium (up to 50% effaced); 2 - Thin (up to 80% effaced); 3 - Very thin / paper-thin (more than 80% effaced)
Cervix position	Cervix position: 0 - Anterior; 1 - Central; 2 - Posterior
Cervix consistency	Cervix consistency: 0 - Soft; 1 - Medium; 2 - Firm
Symphysis fundal height	cm
Sacral promontory reached	Sacral promontory reached: 0 - No; 1 - Yes; 2 - Not assessed<br>**Imputation:** 2
Ischial spines prominent	Ischial spines prominent: 0 - No; 1 - Yes; 2 - Not assessed<br>**Imputation:** 2
Pubic angle admits less than two fingers	Pubic angle admits less than two fingers: 0 - No; 1 - Yes; 2 - Not assessed<br>**Imputation:** 2
Cardiovascular condition	Chronic hypertension, heart disease, obesity, or chronic anaemia: 0 - No; 1 - Yes
Immunity condition	HIV or AIDS: 0 - No; 1 - Yes
Diabetes	Diabetes or gestational diabetes: 0 - No; 1 - Yes
Renal condition	Pyelonephritis or renal disease: 0 - No; 1 - Yes
Lung disease	Lung disease: 0 - No; 1 – Yes<br>**Imputation**: 0
Anaemia	Anaemia: 0 - No; 1 - Yes
Other condition	Other chronic disease, other pregnancy complications, malaria: 0 - No; 1 - Yes

Table B.2. *Follow-up / dynamic features. Bold name indicates that the feature appears modified when compared to the original SELMA dataset. Admission-time imputation refers to variables collected at the time of admission that were missing. Follow-up imputation was resolved through previous follow-up value propagation.*

Name	***Notes***
Contraction ON time	Duration of uterine contractions (seconds).<br>**Admission-time imputation**: first follow-up value.
Contraction OFF time	Time between contractions (seconds).<br>**Admission-time imputation**: first follow-up value.
Cervical dilatation	cm
Maternal Heart rate	bpm
Systolic Blood Pressure	mmHg
Diastolic Blood Pressure	mmHg
Axillary Temperature	degrees Celsius.<br>**Admission-time imputation**: first follow-up value; if also missing, average admission-time value.
Amniotic membranes status	Amniotic membranes status: 0 - Intact; 1 - Ruptured without meconium; 2 - Ruptured with stale meconium; 3 - Ruptured with fresh meconium
Emotional status	Since the last assessment, how much the woman has been bothered by emotional problems such as fear, anxiety, depression, irritability, or sadness? 0 - Not at all; 1 - Slightly; 2 - Moderately; 3 - Quite a bit; 4 – Extremely.<br>**Admission-time imputation**: 0
Labour pain	Since the last assessment, how much the woman has been bothered by labour pain? 0 - Not at all; 1 - Slightly; 2 - Moderately; 3 - Quite a bit; 4 – Extremely.<br>**Admission-time imputation**: 0.
Labour Companionship	Labour Companionship: 0 - No; 1 - Yes
Fetal heart rate	bpm
Fetal movements	Fetal movements observed/felt: 0 - No; 1 - Yes
Fetal presentation	Fetal presentation: 0 - Cephalic; 1 - Breech; 2 - Transverse lie / compound / other
Fetal station	Fetal station: 0 - Above ischial spine; 1 - At ischial spine; 2 - Below ischial spine
Position of fetal head	Position of fetal head: 0 - Occiput Anterior (includes right and left); 1 - Occiput transverse; 2 - Occiput posterior; 3 - Other
Caput Succedaneum	Caput Succedaneum: 0 - None; 1 - Mild; 2 - Moderate; 3 - Severe
Moulding	Moulding: 0 - None; 1 - First degree; 2 - Second degree; 3 - Third degree
Maternal position	Predominant maternal position between assessments: 0 - Upright, sitting, standing, walking, kneeing, squatting, all-4; 1 - Recumbent, semi-recumbent, lateral, supine

Appendix C: Neighbourhood parameters

A grid search was performed over D, L and R to tune values based on performance (revisit eq. **A11** to recall the meaning of these parameters). The parameterisation corresponding to the presented results was $D = 10$ dimensions, $L = 4$ dimensions and $R = 0.5\mu_0$, where μ_0 denotes the average pairwise subject projection distance in the database at $t = 0$.

The values of L and R alone are not very informative regarding the actual number of peers that each test subject is being compared to. Let us consider the final experiment **(step 6 of Figure 4)** where the framework is run for 2121 "test" women, with 6349 women in the projections database.

Figure C.1 plots the histogram of the numbers of peers of all "test" women, at $t = 0$ (left) and considering all follow-ups (right). At $t = 0$, a "test" woman is compared, on average, to 573 "previous" women, corresponding to 9% of all available "training" women. When all follow-ups of all "test" women are accounted for, the average value drops to 370, an expected effect given that the number of available "training" women decreases over time.

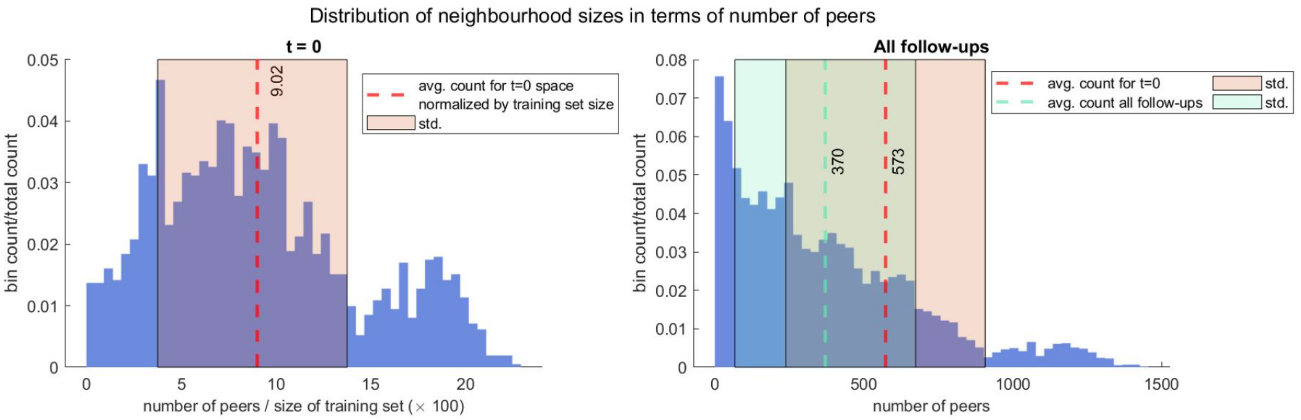

Figure C.1. *Distribution of neighbourhood sizes for step 6 of Figure 4.* Left: at $t = 0$, in terms of percentage of the total number of subjects of the training set. Right: considering all follow-ups, in absolute number.

Appendix D: Other interventions in the MKL space

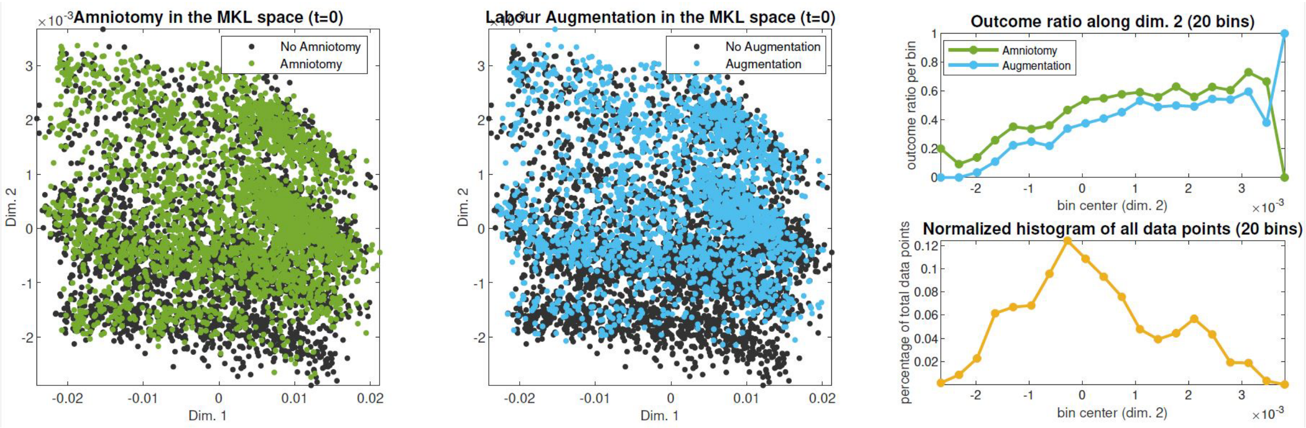

Figure D.1. *Spatial distribution of other interventions in the admission-time MKL space.* Right: amniotomy and labour augmentation rates of occurrence throughout dimension 2, obtained by dividing scatter points in 20 bins along dimension 2 and computing each bin's occurrence rate.

Appendix E: Dimension-variable correlation coefficients

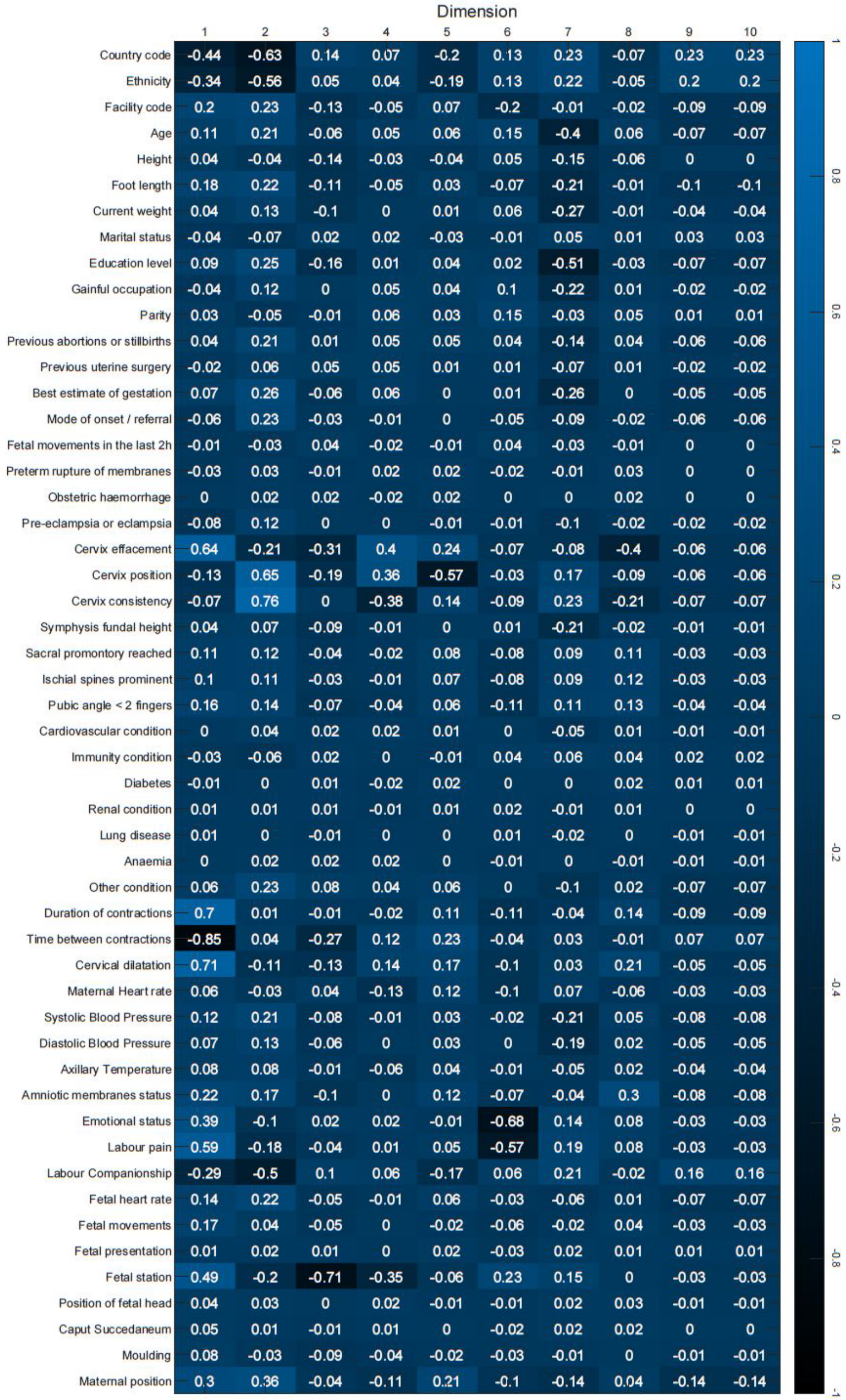

Figure E.1. *Pearson correlation coefficients of the 10 vs. 52 dimension-variable pairs, in the training data admission-time MKL space.*

Appendix F: Performance for latent vs. active phase split

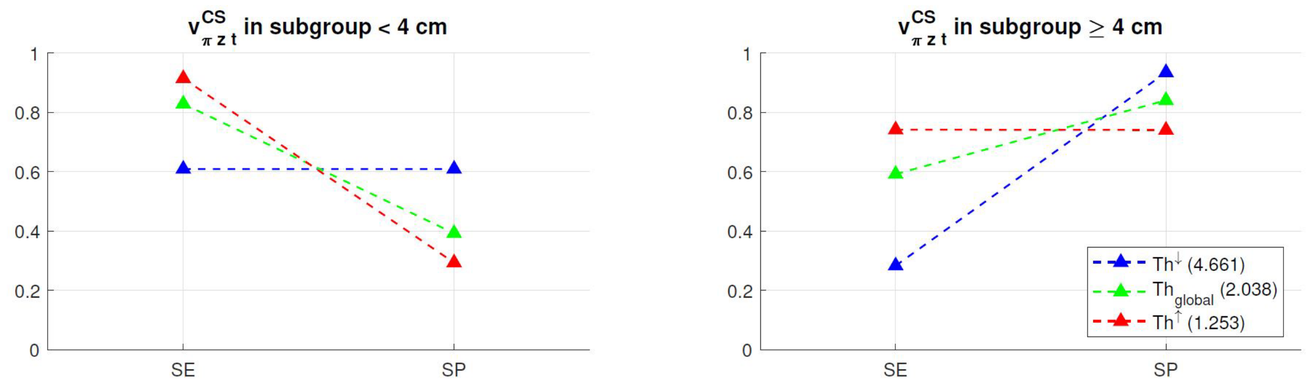

Figure F.1. *Illustration of cut-off value adaptation to optimize performances at the subgroup level*. SE and SP pair for the predictor $v_{\pi z t}^{CS}$ in the "less than 4 cm" (left) and "4 cm and over" (right) subgroups, when the estimated global and subgroup cut-offs are used.

Table F.1. *CS prediction results for "less than 4 cm" and "4 cm and over" subgroups ("↓" and "↑" superscripts, respectively). n = sample size; n_{CS} = number of positive cases; Th = threshold/cut-off; SE = sensitivity; SP = specificity; PPV = positive predictive value; NPV = negative predictive value; AUC = area under the receiver operating characteristic.*

Train *($n^{\downarrow} = 2039$; $n^{\downarrow}_{CS} = 396$; $n^{\uparrow} = 4310$; $n^{\uparrow}_{CS} = 421$)*						
	$Th^{\downarrow}/Th^{\uparrow}$	$SE^{\downarrow}/SE^{\uparrow}$	$SP^{\downarrow}/SP^{\uparrow}$	$PPV^{\downarrow}/PPV^{\uparrow}$	$NPV^{\downarrow}/NPV^{\uparrow}$	$AUC^{\downarrow}/AUC^{\uparrow}$
Alert line	-/-	0.652/0.435	0.502/0.824	0.240/0.211	0.857/0.931	-/-
Action line	-/-	0.434/0.154	0.726/0.958	0.276/0.284	0.842/0.913	-/-
v_{π}^{CS}	0.266/0.179	0.614/0.715	0.616/0.715	0.278/0.214	0.869/0.959	0.670/0.784
$v_{\pi z}^{CS}$	0.583/0.334	0.596/0.713	0.598/0.713	0.263/0.212	0.860/0.958	0.637/0.775
$v_{\pi z t}^{CS}$	4.661/1.253	0.606/0.739	0.609/0.740	0.272/0.235	0.865/0.963	0.646/0.813
Test *($n^{\downarrow} = 708$; $n^{\downarrow}_{CS} = 139$; $n^{\uparrow} = 1413$; $n^{\uparrow}_{CS} = 140$)*						
	$Th^{\downarrow}/Th^{\uparrow}$	$SE^{\downarrow}/SE^{\uparrow}$	$SP^{\downarrow}/SP^{\uparrow}$	$PPV^{\downarrow}/PPV^{\uparrow}$	$NPV^{\downarrow}/NPV^{\uparrow}$	$AUC^{\downarrow}/AUC^{\uparrow}$
Alert line	-/-	0.655/0.443	0.524/0.824	0.251/0.217	0.861/0.931	-
Action line	-/-	0.439/0.143	0.754/0.953	0.303/0.250	0.846/0.910	-
v_{π}^{CS}	0.266/0.179	0.626/0.750	0.643/0.727	0.300/0.232	0.876/0.964	-
$v_{\pi z}^{CS}$	0.583/0.334	0.525/0.700	0.663/0.727	0.275/0.220	0.851/0.957	-
$v_{\pi z t}^{CS}$	4.661/1.253	0.576/0.743	0.634/0.761	0.278/0.255	0.860/0.964	-

Appendix G: Implementation of a prototype decision support system

Based on the ML approach presented in this manuscript, we have developed a prototype decision support system (DSS) called the BCN-SELMA DSS. A snapshot of this prototype is shown in Figure G.1. This prototype, implemented as a web- and cloud-based platform, has been designed with simplicity, scalability, and ease of deployment in mind, which is instrumental for settings with limited resources such as low and middle-income countries (LMICs) where physical storage and computing infrastructure may be lacking.

Once a database of low-dimensional trajectories of “training” patients is available, to handle the management of a new patient, the DSS would require 6 tasks to be sequentially performed:

1. To capture the admission data, the follow-up information while labour progresses, intervention and outcome information.
2. To position (=project) the new patient within the low-dimensional space, learned from the training data.
3. To calculate the expected trajectory for the new patient during labour, as well as to compare the trajectory of the new patient to the ideal one.
4. To provide an estimate of the chance of adverse outcome.
5. To provide an estimate of the need for a certain intervention, that could reverse deviation from the predicted optimal path, at a certain time point in the future during labour progress.
6. To dynamically update the position of the new patient within the low-dimensional space when new measurements become available during labour.

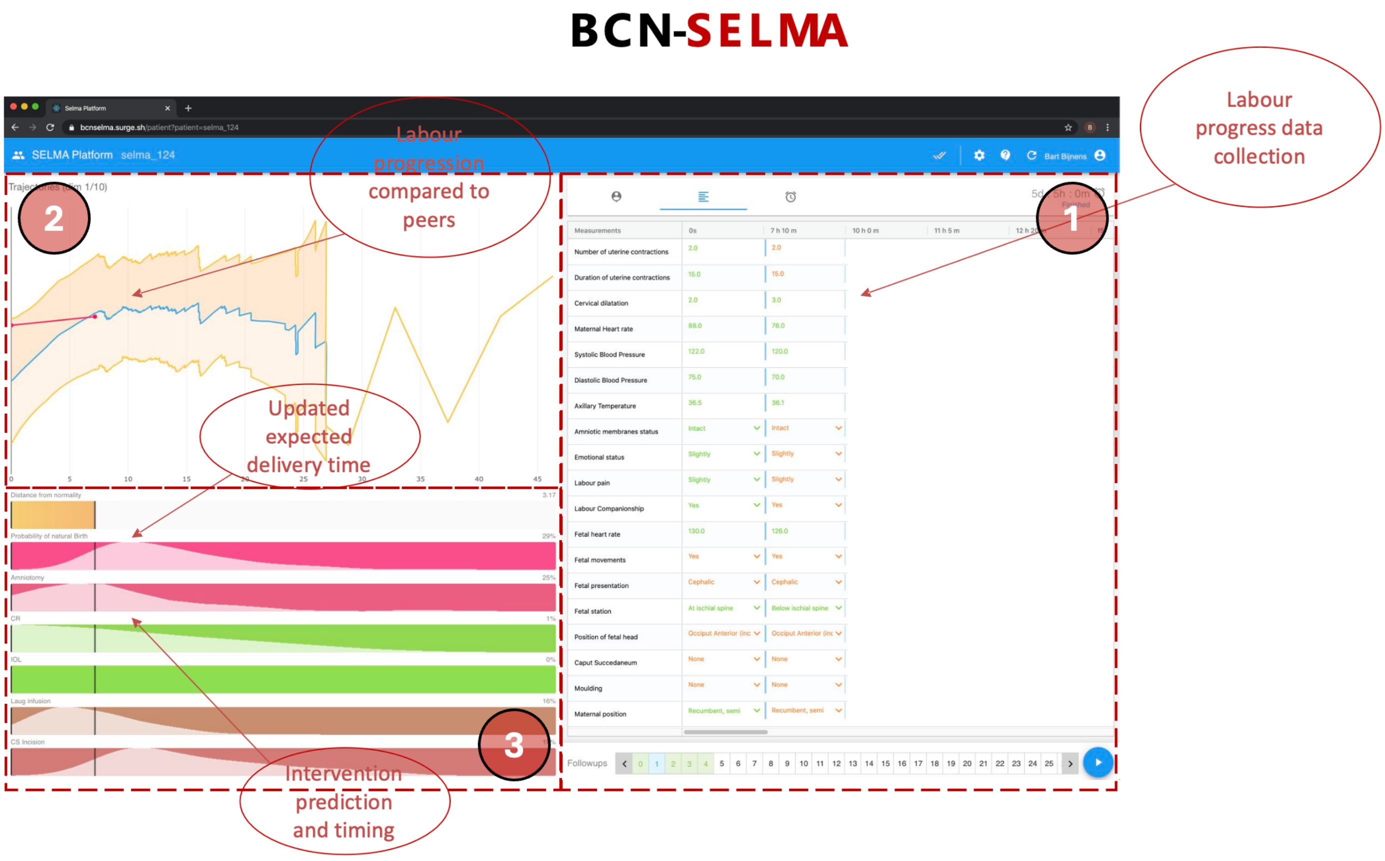

Figure G.1. Snapshot of the BCN-SELMA DSS prototype

To enable the user to input data and obtain all model's outputs enumerated in the tasks above, the prototype is composed of the components indicated by numbers in Figure G.1. Each of these components is explained below:

Component 1: Data entry is performed over a spreadsheet-like form. This sheet has three tabs: one for entering the admission variables, one for entering the dynamic variables during labour progress, and a third one for entering intervention and outcome information.

Component 2: As soon as the required admission information has been entered, the system is triggered to project this information into the learned MKL space. Once the projection of the patient is known, the computation engine will calculate their "ideal" labour progress trajectory, which corresponds to the average trajectory (blue line) and corresponding standard-deviation (orange shadowed region) for those peers that experienced spontaneous, complication-free labours.

Component 3: The top bar shows the distance of the current patient to the "ideal" trajectory at each time-point. The second bar plots the distribution of the time of delivery of the uncomplicated births. The next bars of the web-interface are used to visualise the chance of a certain intervention (in this prototype: amniotomy, cervical ripening, induction of labour, labour augmentation, and caesarean section) and most likely timings.

Practical information

The BCN-SELMA DSS platform is available to the public through the following link. Please note that the interface requires a login. Researchers interested in using the tool should send an email to rocketdev@upf.edu, providing a Google-based email account. Our team will promptly grant free access to the tool.

Once logged in, users can access the platform's help section by clicking the question mark icon located at the upper right of the interface. This section provides several YouTube tutorials, that guide users step-by-step through the various features of the platform, including instructions on how to add a new laboring woman, input information related to the ongoing labour (such as admission data, dynamic variables acquired during labour progress, and details of any interventions and outcome events), and run the algorithm to generate the trajectory and prediction results. It also explains how to query existing patients and download prediction results. Additionally, the authors have included a video as supplemental material to demonstrate a simulated patient interaction on the BCN-SELMA DSS platform. The video begins by duplicating an existing patient profile and then launch projections based on baseline data and successive follow-ups. For the second follow-up, the data is slightly modified to create a less favorable scenario, which consequently shifts the patient's projection further from the expected normal trajectory. Finally, the third follow-up is computed, and the simulated patient profile is saved.

Disclaimer

The BCN-SELMA DSS platform is not certified as a medical device for clinical use. It is intended solely as research software and should not be used for labour assistance or any other clinical purposes.

Appendix H: Availability of Materials

The algorithmic basis of our machine learning model is publicly accessible in both Matlab and Python versions. These resources are available on GitHub at the following repositories: Matlab implementation, and Python implementation.

Additionally, part of the BOLD dataset, which was used to develop our model, has been made publicly available in a prior publication. These materials are accessible through the following links: data set, and data dictionary.

In the spirit of granting accessibility to this work, the SELMA platform discussed in Appendix G remains open to any user who wishes to apply the presented ML model to their labouring data, by simply creating an account. This allows interested researchers from any part of the world to access the model's output in a practical, user-friendly way.

Finally, in their commitment to advancing scientific collaboration and discovery, the authors are open to engaging in further scientific collaborations and, upon reasonable request, can share additional materials under the terms of a scientific collaboration agreement.
